# Diagnostic dilemma between probable cardiac sarcoidosis and non-granulomatous myocarditis: a grand round case report

**DOI:** 10.1093/ehjcr/ytag304

**Published:** 2026-05-07

**Authors:** Tulluru Ravi Sankar, M Ananthakrishna, A Arpudh Michael, P Sirish Chandra Srinath, T Viji Samuel

**Affiliations:** Department of Cardiology, Christian Medical College Hospital, Ida Scudder Road, Vellore, Tamil Nadu 632004, India; Department of Cardiology, Christian Medical College Hospital, Ida Scudder Road, Vellore, Tamil Nadu 632004, India; Department of Cardiology, Christian Medical College Hospital, Ida Scudder Road, Vellore, Tamil Nadu 632004, India; Department of Cardiology, Christian Medical College Hospital, Ida Scudder Road, Vellore, Tamil Nadu 632004, India; Department of Cardiology, Christian Medical College Hospital, Ida Scudder Road, Vellore, Tamil Nadu 632004, India

**Keywords:** Cardiac sarcoidosis, Myocarditis, Steroid-resistant, Heart failure, Immunosuppression, Case report

## Abstract

**Background:**

Cardiac sarcoidosis (CS) can mimic other inflammatory cardiomyopathies, and true steroid resistance is uncommon. Distinguishing granulomatous myocardial involvement from active non-granulomatous myocarditis remains clinically challenging.

**Case summary:**

A 43-year-old woman with biopsy-proven systemic sarcoidosis and dilated cardiomyopathy presented with recurrent decompensated heart failure (HF). Fluorodeoxyglucose positron emission tomography–computed tomography demonstrated diffuse myocardial uptake and was interpreted as probable CS. Despite guideline-directed HF therapy and immunosuppression, she developed progressive HF complicated by renal failure and refractory shock, culminating in death. Post-mortem myocardial biopsy revealed active non-granulomatous myocarditis without granulomas. Rare viral inclusion-like structures consistent with cytomegalovirus were identified in lung tissue, with elevated cytomegalovirus titres; however, immunohistochemistry was negative.

**Discussion:**

This grand round highlights a diagnostic dilemma between probable CS and biopsy-demonstrated non-granulomatous myocarditis. While histology showed myocarditis, co-existing sarcoidosis cannot be definitively excluded due to post-mortem sampling limitations. This case underscores the limitations of imaging-based diagnosis, the potential risks of escalating immunosuppression in the setting of diagnostic uncertainty, and the importance of timely myocardial tissue reassessment, particularly when clinical deterioration occurs despite 2–4 weeks of intensive therapy, within a multidisciplinary framework.

Learning points

- Cardiac sarcoidosis and non-granulomatous myocarditis may present with overlapping clinical and imaging features, limiting diagnostic certainty.
- Fluorodeoxyglucose positron emission tomography–computed tomography -based diagnosis of probable cardiac sarcoidosis does not definitely exclude alternative or concurrent inflammatory cardiomyopathies.
- Clinical deterioration during immunosuppression should prompt early diagnostic reassessment and consideration of endomyocardial biopsy to guide escalation in refractory case management.

## Introduction

Cardiac sarcoidosis (CS) can closely mimic other inflammatory cardiomyopathies, posing substantial diagnostic and therapeutic challenges, particularly when overlapping clinical and imaging features occur in the setting of systemic illness and multiorgan dysfunction. Contemporary guidelines, including the 2024 clinical consensus statement on the management of CS from the Heart Failure Association, the European Association of Cardiovascular Imaging, the European Society of Cardiology Working Group on Myocardial and Pericardial Diseases, and the European Heart Rhythm Association, permit a diagnosis of probable CS in the presence of biopsy-proven extracardiac sarcoidosis with concordant cardiac imaging findings, even without myocardial histologic confirmation.^1,2^ Advanced imaging modalities such as fluorodeoxyglucose positron emission tomography–computed tomography (FDG PET–CT) are central to current diagnostic algorithms but lack disease specificity. Moreover, myocardial histology has limited sensitivity because of the patchy nature of disease involvement.^3,4^

We present a Grand Round case illustrating a diagnostic dilemma between imaging-based probable CS and biopsy-demonstrated non-granulomatous myocarditis. In this patient, extracardiac granulomatous inflammation together with diffuse myocardial fluorodeoxyglucose uptake fulfilled contemporary diagnostic criteria for probable CS, thereby supporting initiation of immunosuppressive therapy under current diagnostic algorithms.^1–4^ This case highlights the limitations of imaging-led diagnosis, the risks of escalating immunosuppression in the setting of diagnostic uncertainty, and the importance of timely multidisciplinary reassessment, including the role of repeat tissue sampling, when clinical deterioration occurs despite guideline-directed therapy.

## Summary figure

**Figure:**
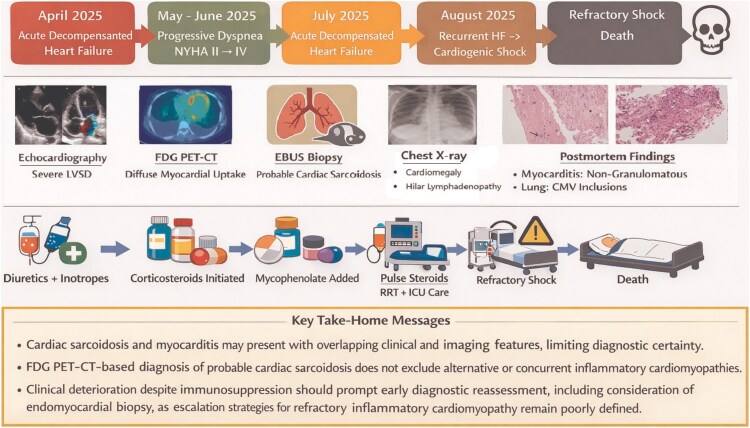
Graphical Summary of the Clinical Course, Diagnostic Evaluation, and Management Pathway

## Case presentation

A 43-year-old woman with no cardiovascular risk factors presented with progressively worsening dyspnoea over 4 months. Three months earlier, she had been admitted to an outside hospital with acute decompensated heart failure (HF), where transthoracic echocardiography revealed severe left ventricular systolic dysfunction (LVSD) with moderate mitral regurgitation. She required intensive care unit (ICU) admission and intravenous dobutamine support. Renal dysfunction was noted (serum creatinine ∼2.0 mg/dL) and subsequent imaging revealed bilateral renal cortical scarring on ultrasonography, suggesting underlying chronic kidney disease, possibly related to reflux nephropathy or prior asymptomatic infections. She was advised for further evaluation at a tertiary-care centre.

Although she improved transiently, her symptoms progressed from New York Heart Association functional class II to IV over the subsequent two months, accompanied by orthopnoea and paroxysmal nocturnal dyspnoea. She denied chest pain, fever, haemoptysis, sputum, wheeze, photosensitivity, rash, haematuria, or frothy urine. There was no history of tobacco, alcohol, or nephrotoxic or cardiotoxic drug exposure. Her past medical history was otherwise unremarkable, with no diabetes mellitus, hypertension, coronary artery disease, tuberculosis, or prior cardiac disease. Surgical history included childhood appendicectomy, and obstetric history included one uncomplicated pregnancy with normal vaginal delivery 15 years earlier. Family history was non-contributory.

On presentation to our institution, she was dyspneic at rest. She measured 163 cm in height and weighed 58.6 kg (body mass index 22.1 kg/m^2^), with blood pressure 94/64 mmHg and heart rate 84 beats/min. Physical examination revealed elevated jugular venous pressure, bilateral basal crepitations, and bilateral 2+ pedal oedema. Cardiac auscultation revealed a soft first heart sound, a normal second heart sound, and a grade 2/6 holosystolic murmur at the apex. There was no rash, lymphadenopathy, cyanosis, or clubbing.

Given the presentation of severe LVSD, differential diagnoses included inflammatory and non-ischaemic cardiomyopathies.^1–4^ CS was strongly suspected given bilateral mediastinal lymphadenopathy (*Figure 1A*) and elevated serum angiotensin-converting enzyme levels. Active myocarditis, including viral or immune-mediated aetiologies, was considered because of the subacute course and systemic inflammation. Genetic dilated cardiomyopathy was considered and evaluated. Giant cell myocarditis was deemed less likely in the absence of fulminant conduction disease or eosinophilia. Ischaemic cardiomyopathy was unlikely given absent risk factors and non-ischaemic echocardiographic features (*Figure 2*). Infiltrative cardiomyopathies and stress-induced cardiomyopathy were considered less likely given imaging findings and the chronic progressive course. Concomitant renal dysfunction and systemic illness further complicated early diagnostic clarity.

**Figure 1:**
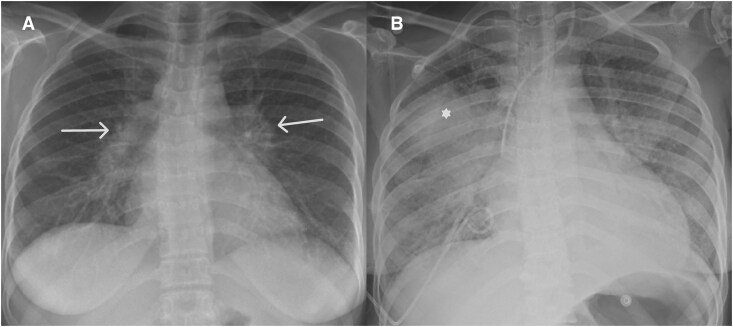
Chest radiographs at initial presentation and late hospital course. (*A*) Chest radiograph obtained at initial presentation (3 June 2025) demonstrating bilateral hilar lymphadenopathy (arrows). (*B*) Chest radiograph obtained one day prior to death (28 August 2025) demonstrating patchy pulmonary opacification, more prominent in the right upper lung zone (asterisk).

**Figure 2:**
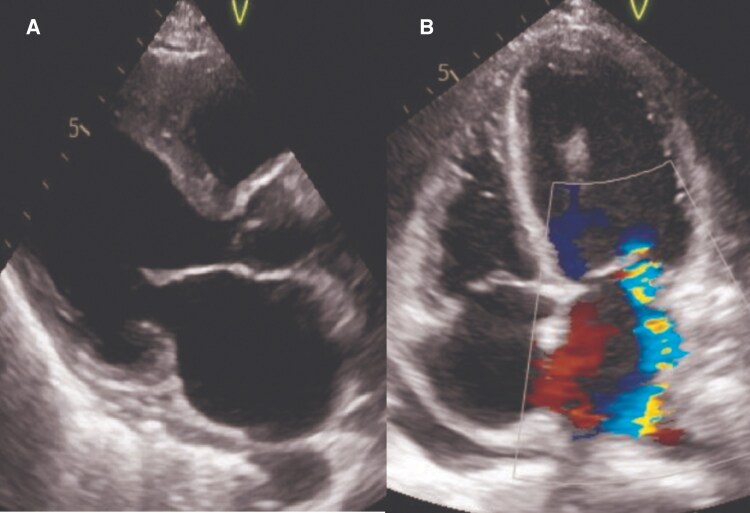
Transthoracic echocardiography. (*A*) Parasternal long-axis view demonstrating a markedly dilated left ventricle. (*B*) Apical four-chamber view demonstrating left ventricular dilatation with eccentric mitral regurgitation.

Electrocardiography showed sinus rhythm with low-voltage QRS complexes and non-specific ST/T changes (*Figure 3*), with non-sustained ventricular tachycardia documented during ICU monitoring. Transthoracic echocardiography revealed severe LVSD with Left ventricular ejection fraction of 20%, global hypokinesia, grade III diastolic dysfunction, moderate secondary mitral regurgitation, mild tricuspid regurgitation, pulmonary hypertension (RVSP 55–57 mmHg), and borderline right ventricular dysfunction. Chest radiography demonstrated cardiomegaly, pulmonary congestion, and hilar lymphadenopathy, while serial imaging showed progressive bilateral infiltrates paralleling worsening hypoxaemia and clinical deterioration (*Figure 1B*). Computed tomography pulmonary angiography excluded pulmonary embolism but demonstrated upper-lobe–predominant perihilar consolidations and bilateral pleural effusions (*Figure 4*). Laboratory investigations revealed markedly elevated *n*-terminal pro-B-type natriuretic peptide levels (>35 000 pg/mL), fluctuating renal dysfunction, progressive thrombocytopenia, anaemia, and intermittent transaminitis. During the ICU course, blood cultures grew Klebsiella species, coinciding with worsening cytopenias and haemodynamic instability. FDG PET–CT demonstrated diffuse myocardial FDG uptake with metabolically active mediastinal lymph nodes (*Figure 5*). Endobronchial ultrasound-guided lymph node biopsy revealed non-necrotizing granulomatous inflammation, confirming systemic sarcoidosis. Infectious and autoimmune evaluations were otherwise negative.

**Figure 3:**
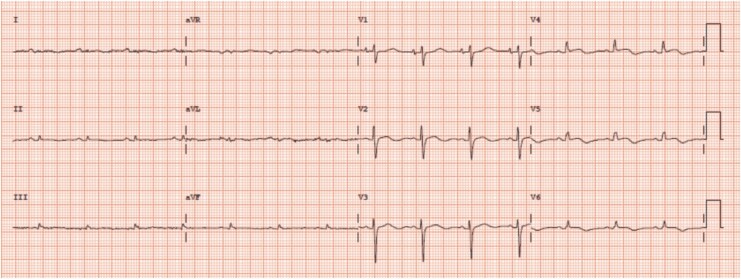
Electrocardiogram at initial presentation. Twelve-lead electrocardiogram demonstrating sinus rhythm with low-voltage QRS complexes and non-specific ST–T wave changes.

**Figure 4:**
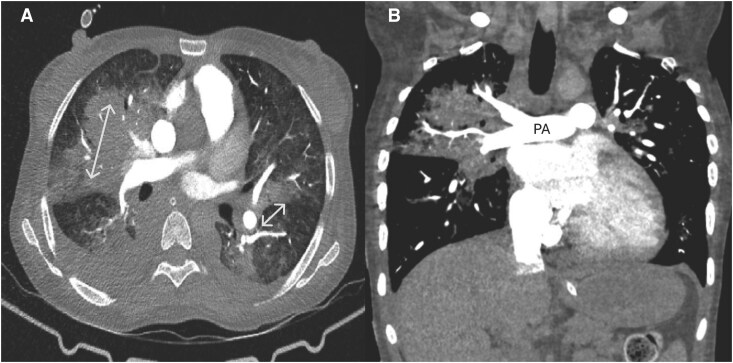
Computed tomography pulmonary angiogram of the chest. Computed tomography pulmonary angiogram demonstrating diffuse interlobular septal thickening with perihilar and broncho centric pulmonary opacities (upper-lobe predominant, right greater than left) (double-headed arrows), along with bilateral pleural effusions (right greater than left). (*A*) Axial image. (*B*) Coronal image. PA = pulmonary artery.

**Figure 5:**
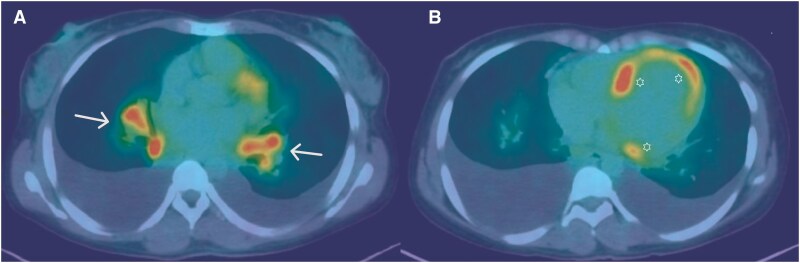
Fluorodeoxyglucose positron emission tomography–computed tomography of the chest. (*A*) Axial FDG PET–CT image demonstrating metabolically active enlarged mediastinal lymph nodes (arrows). (*B*) Axial FDG PET–CT image demonstrating increased myocardial fluorodeoxyglucose uptake, consistent with active myocardial inflammation (asterisks).

The patient was managed with a multidisciplinary approach involving cardiology, pulmonology, nephrology, infectious diseases, dermatology, and haematology teams. Acute HF was initially treated with intravenous loop diuretics and strict fluid balance. Guideline-directed medical therapy (GDMT) was limited by hypotension and renal dysfunction; β-blockers and vasodilators were trialled twice but discontinued, while Dapagliflozin was continued as tolerated. Inotropic support included a single course of dobutamine during the initial admission and two dopamine infusions and one course of levosimendan during the second admission, with only transient improvement. In view of biopsy-proven extracardiac sarcoidosis and FDG PET–CT evidence of myocardial inflammation, immunosuppression was initiated with oral prednisolone (0.5 mg/kg/day).^3,4^ Mycophenolate mofetil was added after normalization of liver function tests.^5^ Progressive clinical deterioration prompted escalation to pulse corticosteroid therapy with intravenous dexamethasone followed by methylprednisolone, with subsequent transition to high-dose oral steroids after multidisciplinary team discussion.^6–8^ Worsening renal failure and refractory congestion necessitated renal replacement therapy (RRT).

New-onset thrombocytopenia, likely secondary to sepsis, drug toxicity, or viral infection, resulted in petechiae and ecchymoses over the trunk and extremities and improved without transfusion. A skin biopsy of persistent papulonodular lesions showed non-specific inflammatory changes without granulomas, suggesting a reactive process to drugs or infection, rather than cutaneous sarcoidosis. No specific dermatologic intervention was advised by dermatologist. The patient subsequently developed oral candidiasis following corticosteroid therapy, which was treated with systemic fluconazole. During the late ICU course, she developed culture-proven *Klebsiella* sepsis, associated with further clinical deterioration. Despite broad-spectrum antimicrobial therapy, only transient improvement was observed. Recurrent non-sustained ventricular arrhythmias were documented on continuous telemetry and were managed medically. Implantable cardioverter-defibrillator implantation was recommended but deferred due to sepsis, thrombocytopenia, and haemodynamic instability.

Despite aggressive HF management, escalating immunosuppression, RRT, and treatment of sepsis, the patient developed refractory cardiogenic and distributive shock with multiorgan failure. Pulmonary embolism was excluded during acute haemodynamic collapse. Despite maximal supportive care, including vasopressors, inotropes, ventilatory support, and dialysis, she died during the hospitalization. Post-mortem endomyocardial biopsy (EMB) revealed active non-granulomatous myocarditis with myocyte injury and interstitial fibrosis, without evidence of sarcoid granulomas. Rare cytomegalovirus-like inclusion bodies were identified in lung tissue, and subsequent testing demonstrated elevated circulating cytomegalovirus titres; although, immunohistochemistry for cytomegalovirus was negative (*Figure 6*). Clinical exome sequencing, sent during the ICU stay, was reported posthumously and showed no pathogenic variants associated with cardiomyopathy. Integrated Clinical Timeline and Key Decision Points are summarized in *Table 1*.

**Figure 6:**
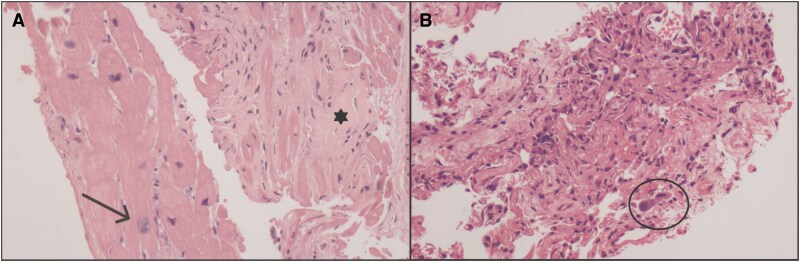
Post-mortem histopathological findings in myocardial and pulmonary tissue (haematoxylin and eosin stain; original magnification ×200 for both panels). (*A*) Post-mortem myocardial biopsy demonstrating hypertrophic myocardial fibres (arrow) with interstitial fibrosis (asterisk). (*B*) Post-mortem lung biopsy demonstrating viral inclusion-like cytopathic changes, morphologically consistent with cytomegalovirus-like inclusions (circle).

**Table 1:** Integrated clinical timeline and key decision points

Date/phase	Clinical course	Key diagnostic findings	Major management decisions
3–5 Apr 2025 (initial hospitalization)	Acute decompensated HF with cardiogenic shock	TTE: severe LV dysfunction, moderate MR	ICU care; IV diuretics; Dobutamine support
Apr–Jun 2025 (outpatient progression)	Progressive dyspnoea (NYHA II → IV)	Persistent renal dysfunction	Optimization of GDMT
20 Jun−9 Jul 2025 (1st tertiary admission)	Recurrent decompensated HF	FDG PET–CT: diffuse myocardial uptake EBUS LN biopsy: non-necrotizing granulomas	Diagnosis of probable cardiac sarcoidosis; initiation of corticosteroids
Mid-Jul 2025 (early post-discharge course)	GDMT intolerance; persistent congestion	Renal venous congestion (VEXUS)	Diuretic uptitration; addition of mycophenolate
9–20 Aug 2025 (2nd admission)	Acute decompensated HF with rash and worsening renal function	NT-proBNP >35 000 pg/mL; worsening cytopenias	CICU admission; inotropes (dopamine, levosimendan); non-invasive ventilation
21–23 Aug 2025 (ICU deterioration)	Progressive HF, acute kidney injury, bicytopenia	Rising creatinine; ventricular arrhythmias	Renal replacement therapy initiation; cessation of potential offending agents
24–28 Aug 2025 (escalation phase)	Sepsis with persistent shock; suspected steroid non-responsiveness	Blood culture: Klebsiella species Persistent pulmonary oedema Worsening cytopenias	Antibiotics uptitration, Vasopressor escalation, Pulse steroids (IV dexamethasone → methylprednisolone); Renal replacement therapy
29 Aug 2025 (terminal event)	Refractory cardiogenic shock leading to cardiac arrest at 15:15	CT pulmonary angiography excluded pulmonary embolism	Escalation of inotropes Cardiac arrest and death
Post-mortem	—	EMB: active non-granulomatous myocarditis. Lung tissue: CMV-like inclusion bodies with negative IHC and elevated CMV titres. Clinical exome sequencing: No pathogenic variants	Diagnostic discordance with imaging-based classification

CICU, cardiac intensive care unit; CMV, cytomegalovirus; CT, computed tomography; EBUS, endobronchial ultrasound; EMB, endomyocardial biopsy; FDG PET–CT, fluorodeoxyglucose positron emission tomography–computed tomography; GDMT, guideline-directed medical therapy; HF, heart failure; ICU, intensive care unit; IHC, immunohistochemistry; IV, intravenous; LV, left ventricle; MR, mitral regurgitation; NT-proBNP, *n*-terminal pro-B-type natriuretic peptide; NYHA, New York Heart Association; TTE, transthoracic echocardiography; VEXUS, venous excess ultrasound score.

## Discussion

In this patient, the presence of non-necrotizing granulomas on extracardiac lymph node biopsy, together with diffuse myocardial fluorodeoxyglucose uptake, fulfilled contemporary consensus criteria for probable CS (*Table 2*). This diagnostic approach aligns with current recommendations, including recent consensus statements from the Heart Failure Association of the European Society of Cardiology and the American Heart Association.^1–4,9^ However, progressive clinical deterioration despite escalating immunosuppression highlighted the diagnostic uncertainty that may persist even when guideline-based criteria are fulfilled. Fluorodeoxyglucose uptake reflects inflammatory activity but lacks specificity and does not distinguish between granulomatous and non-granulomatous inflammation, which may lead to diagnostic overlap. This case illustrates how reliance on imaging-based diagnostic pathways, in the absence of definitive myocardial histology, may obscure alternative or concurrent inflammatory cardiomyopathies and complicate therapeutic decision-making.

**Table 2:** Diagnostic criteria for cardiac sarcoidosis and application to the present case

Diagnostic component	Guideline requirement	Findings in this patient	Fulfilled (Yes/No)
Extracardiac histology	Histological evidence of non-caseating granulomas in extracardiac tissue	EBUS-guided lymph node biopsy showing non-necrotizing granulomas	✅ Yes
Cardiac imaging (FDG PET–CT)	Evidence of myocardial inflammation (focal or diffuse FDG uptake)	Diffuse myocardial FDG uptake with metabolically active mediastinal lymph nodes	✅ Yes
Clinical cardiac involvement	Unexplained cardiomyopathy, arrhythmias, or conduction abnormalities	Severe LV dysfunction with heart failure; ventricular arrhythmias	✅ Yes
Exclusion of alternative diagnoses (initially)	No better explanation at time of diagnosis	No definitive alternative diagnosis at initial presentation	✅ Yes
Endomyocardial biopsy	Not mandatory for probable Cardiac Sarcoidosis	Not performed during index admission due to clinical instability	❌ Not performed

EBUS, endobronchial ultrasound; FDG PET–CT, fluorodeoxyglucose positron emission tomography–computed tomography; LV, left ventricle.

Corticosteroids remain the first-line therapy for clinically manifest CS, with steroid-sparing agents reserved for refractory disease or intolerance. Although a ‘hit-hard and early’ therapeutic approach has been proposed for severe sarcoidosis, advocating earlier use of steroid-sparing agents or biologics to prevent irreversible organ damage, supporting evidence is largely observational, and inter-centre variability in immunosuppressive practice persists.^5^ True steroid resistance in CS is uncommon and may instead reflect irreversible myocardial injury, alternative inflammatory cardiomyopathies, or superimposed infection.^7,8^ In such scenarios, further escalation of immunosuppression may be ineffective or harmful. In the present case, immunosuppressive therapy was initiated for probable CS in accordance with contemporary HF association guidelines; however progressive deterioration despite treatment raised concern for alternative inflammatory aetiologies or superimposed infection rather than true steroid resistance.

EMB has limited sensitivity for CS because of sampling variability. Current guidelines recommend EMB in patients with new-onset cardiogenic shock of unclear aetiology, persistent clinical deterioration or when alternative diagnoses such as myocarditis are suspected. In this case, EMB was deferred during the index admission because of haemodynamic instability, thrombocytopenia, and evolving multiorgan dysfunction, which posed a high procedural risk. Cardiac magnetic resonance imaging was similarly not feasible, underscoring the practical limitations of advanced imaging in critically ill patients. The post-mortem myocardial examination demonstrated active non-granulomatous myocarditis, highlighting potential discordance between imaging-based diagnosis and limited myocardial histologic sampling. It should be noted that post-mortem evaluation was limited to targeted myocardial sampling rather than comprehensive examination of the entire heart; therefore, the possibility of sampling error cannot be excluded, and focal granulomatous involvement consistent with CS may have been missed. Accordingly, these findings should be interpreted with caution, and coexistence of CS and non-granulomatous myocarditis cannot be definitively excluded. These findings highlight the limitations of both imaging and tissue sampling in inflammatory cardiomyopathies, particularly in the context of patchy myocardial involvement. In retrospect, earlier consideration of EMB may have been appropriate after lack of clinical improvement despite 2–4 weeks of corticosteroid therapy. Although it remains uncertain whether earlier biopsy would have altered the clinical outcome, histologic clarification might have influenced therapeutic decision-making, including reconsideration of immunosuppressive escalation and evaluation for alternative aetiologies. This case underscores the importance of timely reassessment and individualized decision-making regarding EMB in critically ill patients.

Rare cytomegalovirus-like inclusions were identified in post-mortem lung tissue, together with elevated circulating cytomegalovirus titres despite negative immunohistochemistry. The discrepant cytopathic changes suggest possible systemic viral reactivation in the setting of immunosuppression, although alternative explanations such as infection with low viral load, focal sampling, other viral aetiologies with similar morphology, drug-related cytopathic changes, or post-mortem autolysis cannot be excluded.^10–12^ Although Cytomegalovirus infection has been associated with myocarditis and systemic manifestations such as cytopenias, renal dysfunction, a causal relationship with the observed myocardial inflammation cannot be established in this case. Accordingly, the pulmonary and serologic findings are best interpreted as incidental or contributory to systemic illness rather than a primary driver of myocardial pathology.

The interpretation of non-granulomatous myocarditis in this context warrants careful consideration. The observed myocardial inflammation may represent a primary inflammatory myocarditis underlying the initial presentation. Alternatively, it may reflect a secondary process related to prolonged immunosuppression, systemic infection, or critical illness, all of which may contribute to myocardial injury and inflammatory changes. A mixed process, including coexistence of CS with superimposed myocarditis, also remains plausible, given the limitations of myocardial sampling. In the absence of comprehensive myocardial evaluation and definitive etiologic markers, it is not possible to determine whether myocarditis was the primary driver of the clinical course or a secondary phenomenon. Although both CS and fulminant myocarditis may initially warrant immunosuppressive therapy, the intensity, duration, and adjunctive agents differ significantly. This distinction has important therapeutic implications, particularly in guiding escalation or de-escalation of immunosuppressive therapy. Given the substantial haematologic and renal toxicity of antiviral agents, empiric therapy was unlikely to confer meaningful benefit in the setting of advanced HF, multiorgan dysfunction, and diagnostic uncertainty.

Collectively, this case does not represent a deviation from established guidelines but rather highlights the diagnostic uncertainty that may persist despite adherence to consensus-based criteria and the complexity of managing steroid-refractory inflammatory cardiomyopathy. Early histologic clarification may influence the selection of second-line immunosuppressants, consideration of antiviral therapy, and candidacy for advanced HF therapies. It underscores the importance of timely reassessment, multidisciplinary decision-making, and consideration of myocardial tissue diagnosis in patients with suspected inflammatory cardiomyopathy who deteriorate despite guideline-directed therapy. Diagnosis and management in inflammatory cardiomyopathy should remain dynamic and responsive to evolving clinical data.

## Patient’s perspective

Although a direct patient perspective was unavailable due to the patient’s clinical deterioration and subsequent death, her family remained closely engaged throughout the prolonged ICU course. They experienced significant emotional distress while witnessing her rapid decline despite escalation of guideline-directed therapies. This case highlights the importance of transparent communication with families regarding the uncertainties inherent in diagnosis, the limitations of imaging-based evaluation, and the potential risks of empiric immunosuppression, particularly in the setting of multiorgan dysfunction.

## Data Availability

All relevant data supporting the findings of this study are available within the article.

## Lead author biography

I am currently working as an Associate Professor in the Department of Cardiology at Christian Medical College, Vellore, India. I completed my DM in Cardiology from the same institution, where I developed a focused interest in complex coronary interventions. My academic interests include clinical research, interventional outcomes, and procedural optimization in challenging coronary scenarios.

## Notes

**Consent:** Written informed consent was obtained from the patient’s next of kin for publication of this case and accompanying images where appropriate.

## Specialities other than cardiology involved

Electrophysiology, Pulmonology, Nephrology, Infectious Diseases, Pathology, Haematology, Dermatology

## References

[1] Cheng RK, Kittleson MM, Beavers CJ, Birnie DH, Blankstein R, Bravo PE, et al Diagnosis and management of cardiac sarcoidosis: a scientific statement from the American Heart Association. Circulation 2024;149:e1197–e1216. 38634276 10.1161/CIR.0000000000001240

[2] Sharma R, Kouranos V, Cooper LT, Metra M, Ristic A, Heidecker B, et al Management of cardiac sarcoidosis: a clinical consensus statement of the heart failure association, the European association of cardiovascular imaging, the ESC working group on myocardial and pericardial diseases, and the European heart rhythm association of the ESC. Eur Heart J 2024;45:2697–2726.38923509

[3] Shah HH, Zehra SA, Shahrukh A, Waseem R, Hussain T, Hussain MS, et al Cardiac sarcoidosis: a comprehensive review of risk factors, pathogenesis, diagnosis, clinical manifestations, and treatment strategies. Front Cardiovasc Med 2023;10:1156474. 37273881 10.3389/fcvm.2023.1156474 PMC10235776

[4] Lehtonen J, Uusitalo V, Pöyhönen P, Mäyränpää MI, Kupari M. Cardiac sarcoidosis: phenotypes, diagnosis, treatment, and prognosis. Eur Heart J 2023;44:1495–1510. 36924191 10.1093/eurheartj/ehad067 PMC10149532

[5] Vorselaars ADM, Culver DA. Hit-hard and early versus step-up treatment in severe sarcoidosis. Curr Opin Pulm Med 2022;28:461–467. 35869744 10.1097/MCP.0000000000000906 PMC9451911

[6] El Jammal T, Jamilloux Y, Gerfaud-Valentin M, Valeyre D, Sève P. Refractory sarcoidosis: a review. Ther Clin Risk Manag 2020;16:323–345. 32368072 10.2147/TCRM.S192922 PMC7173950

[7] Bhimani S, Rojulpote C, Deshpande Y, Maligireddy AR. Cardiac sarcoidosis: the role of steroid therapy in managing myocardial inflammation and arrhythmic risks. World J Cardiol 2025;17:107637. 41356578 10.4330/wjc.v17.i11.107637 PMC12678888

[8] Grabowska O, Martusewicz-Boros MM, Piotrowska-Kownacka D, Wiatr E. Steroid-resistant cardiac sarcoidosis successfully managed with methotrexate: a case report. Sarcoidosis Vasc Diffuse Lung Dis 2018;35:178–181. 32476900 10.36141/svdld.v35i2.6554 PMC7170091

[9] Manabe O, Oyama-Manabe N, Aikawa T, Tsuneta S, Tamaki N. Advances in diagnostic imaging for cardiac sarcoidosis. J Clin Med 2021;10:5808. 34945105 10.3390/jcm10245808 PMC8704832

[10] Solomon IH, Hornick JL, Laga AC. Immunohistochemistry is rarely justified for the diagnosis of viral infections. Am J Clin Pathol 2017;147:96–104. 28007703 10.1093/ajcp/aqw198

[11] Eyzaguirre E, Collins BT. Viral infections (cytomegalovirus, herpesvirus, and others). In: Cibas ES, Ducatman BS, eds. Diagnostic Pathology: Cytopathology. 2nd ed. Philadelphia: Elsevier; 2018. p114–115.

[12] Saraca LM, Lazzari L, Di Giuli C, Lavagna A, Mezzetti P, Bovelli D, et al Cytomegalovirus myocarditis in a patient with systemic lupus erythematosus successfully treated with ganciclovir. IDCases 2018;12:4–6. 29850400 10.1016/j.idcr.2018.02.008 PMC5966621

